# The cGAS-STING pathway in COPD: targeting its role and therapeutic potential

**DOI:** 10.1186/s12931-024-02915-x

**Published:** 2024-08-07

**Authors:** Kexin Liao, Fengshuo Wang, Chenhao Xia, Ze Xu, Sen Zhong, Wenqi Bi, Jingjing Ruan

**Affiliations:** 1 First Clinical Medical College, Anhui Medical University, Hefei, 230022 People’s Republic of China; 2 Department of Respiratory and Critical Care Medicine, The First Affiliated Hospital of Anhui Medical University, Hefei, 230022 People’s Republic of China; 3 College of Pharmacy, Anhui Medical University, Hefei, 230022 People’s Republic of China

**Keywords:** cGAS-STING pathway, COPD, Agonists, Therapeutic potential

## Abstract

Chronic obstructive pulmonary disease(COPD) is a gradually worsening and fatal heterogeneous lung disease characterized by airflow limitation and increasingly decline in lung function. Currently, it is one of the leading causes of death worldwide. The consistent feature of COPD is airway inflammation. Several inflammatory factors are known to be involved in COPD pathogenesis; however, anti-inflammatory therapy is not the first-line treatment for COPD. Although bronchodilators, corticosteroids and roflumilast could improve airflow and control symptoms, they could not reverse the disease. The cyclic GMP-AMP synthase-stimulator of interferon genes (cGAS-STING) signaling pathway plays an important novel role in the immune system and has been confirmed to be a key mediator of inflammation during infection, cellular stress, and tissue damage. Recent studies have emphasized that abnormal activation of cGAS-STING contributes to COPD, providing a direction for new treatments that we urgently need to develop. Here, we focused on the cGAS-STING pathway, providing insight into its molecular mechanism and summarizing the current knowledge on the role of the cGAS-STING pathway in COPD. Moreover, we explored antagonists of cGAS and STING to identify potential therapeutic strategies for COPD that target the cGAS-STING pathway.

## Introduction

COPD is a progressive and debilitating respiratory disease that affects millions of people worldwide and poses a considerable medical and financial burden [1, 2]. Traditionally, COPD is considered as an inflammatory response elicited by cigarette smoking(CS) in older males [3]. In addition, other factors, such as air pollution, occupational particles and aging, have also been found to trigger lung inflammation, with COPD subsequently accompanied by inflammation [2].

Pattern recognition receptors (PRRs) are a significant component of the innate immune system and constitute the first line of defense in organisms. As a member of the PPR family, the cGAS protein acts as an innate nucleic acid sensor recognizing exogenous DNA generated by viral or bacterial infection or in the cytoplasm and converts ATP and GTP into 2’3’-cyclic GMP-AMP (cGAMP), which can be used to monitor pathogen infection or cellular stress [4]. cGAMP binds to the adapter protein stimulator of interferon genes (STING) localized at the endoplasmic reticulum (ER) membrane [5] and initiates a downstream immune response. Increasing research suggests that the cGAS-STING pathway plays an important role in the development of many diseases through its involvement in autoimmunity, cellular senescence and anti-inflammation [6].

The Global Initiative of Obstructive Lung Disease (GOLD) has suggested guidelines for COPD management. However, symptomatic treatment involving bronchodilators continues to be the mainstay in COPD management, despite the understanding of inflammation as a key driver of COPD progression. There is currently no cure for COPD. Recent efforts have tended to focus on the molecular mechanisms underlying COPD to explore therapeutic targets for COPD. Studies have shown that cGAS-STING contributes to COPD, especially under exposure to cigarette smoking [7] or air pollutants including silica [8] and PM 2.5 [9]. Moreover, targeting the cGAS-STING pathway can circumvent cellular senescence [10, 11], which has also been shown to contribute to the accelerated aging process in COPD patients [12]. Delving into the structure and function of the cGAS-STING pathway may enable the development of selective small-molecule inhibitors to manage the inflammation associated with COPD. In this review, we discussed the role of the cGAS-STING pathway in the pathogenesis of COPD, as well as antagonists of this pathway, with a focus on its therapeutic potential for COPD. Our aim is to contribute to the optimization of fundamental therapies for COPD, ultimately improving patient prognosis.

## Inflammation-associated mechanisms in the pathogenesis of COPD

Airway inflammation is a consistent feature of COPD and plays an important role in the disease pathogenesis, progression and mortality [2, 13]. Inflammation has many manifestations. In this paragraph, we describe neutrophil-associated and eosinophil-associated inflammation in COPD as well as some relevant inflammatory signaling pathways.

### Neutrophil-associated airway inflammation in COPD

Neutrophil inflammation is the key inflammatory phenotype in the pathogenesis of COPD, with increased neutrophils in sputum and blood being a characteristic feature of all COPD patients. Studies have reported that neutrophil count is a marker of COPD severity and patients with higher sputum neutrophil percentages have greater dyspnea scores [2, 14, 15]. When stimulated by inflammation, neutrophils leave the circulation to congregate in lungs. The aggregation of neutrophils produces a large amount of reactive oxygen species (ROS), which can destroy lung tissues [16]. Moreover, neutrophils produce the inflammatory factor IL-6, which induces the production of elastase and oxygen free radicals, thereby increasing pulmonary vascular permeability and exacerbating lung tissue destruction [17]. Neutrophils accumulate in the airways of COPD patients [18] and can secrete serine proteases including matrix metalloproteinase (MMP) and neutrophil elastase (NE) [19]. MMP is significantly increased in patients with COPD and destroys the structural components of extracellular matrix (ECM), contributing to alveolar destruction [20]. In animal models, dominant-negative MafB was shown to downregulated MMP, thereby suppressing porcine pancreatic elastase-induced emphysema [21]. NE is a neutrophil-derived serine proteinase and has proven to be involved in lung damage. A study revealed that NE deficiency in mice protects them from emphysema after exposure to cigarette smoke (CS) [22]. The underlying mechanism may be that NE can also degrade the structural components of ECM and cooperate with MMPs to amplify the degradation [23]. In addition, NE is effective in stimulating mucus secretion from submucosal glands and thrush cells, leading to airway obstruction [24]. All these findings indicate the contribution of neutrophil inflammation to the development of COPD.

### Eosinophilic-associated airway inflammation in COPD

Although neutrophil-associated COPD is the most common inflammatory phenotype, it has been recognized that eosinophils may also be involved in the inflammatory response in COPD. Approximately 10-40% of COPD patients demonstrate increased eosinophilic inflammation in the sputum or blood [25]. Eosinophilic airway inflammation occurs in COPD exacerbations. Clinical research has shown that patients with high eosinophil count but a low percentage of macrophages exhibit the greatest decline in lung function during an exacerbation and a greater exacerbation frequency. This group of patients has persistent eosinophilic inflammation due to defective macrophage efferocytosis, which contributes to the severity of the disease [26]. Like in asthma, recruitment of eosinophils to the airway in COPD is mediated via CCR3 chemokines, which play a critical role together with other eosinophil chemoattractants, such as prostaglandin (PG)D2 [27, 28]. Inflammatory cues prompt the recruitment of eosinophils into the lungs, where the secretion of a variety of chemokines (e.g., CCL5, CCL11, CCL13), cytokines (e.g., IL-2, IL-3, IL-4, IL-5, IL-10, IL-12, IL-13, IL-16, IL-25) and cytotoxic granular products (major basic protein, eosinophil cationic protein, eosinophil peroxidase, eosinophil-derived neurotoxin) contributes to inflammation [29]. An increase in eosinophilic inflammation in peripheral blood and sputum samples from COPD patients is associated with an increased risk of severe deterioration in the future [30]. However, the etiology of eosinophilic inflammation in COPD is not completely understood.

### Inflammation-associated pathways in COPD

The pathogenesis of COPD involves the activation of diverse inflammatory pathways. The NF-κB pathway is activated by the ubiquitination of IκB [31]. As a result, NF-κB is released from the NF-κB/IκB complex and are able to bind to target genes, thereby initiating the expression of target genes, such as TNF-α and IL-1, and causing an inflammatory response [32]. In addition, the inhalation of ozone and cigarette smoke results in the migration of neutrophils into the lungs to generate ROS, which is another factor in the activation of NF-κB [33]. A study in mouse models of COPD demonstrated that NF-kB pathway is essential for inflammation in smoking-induced bronchiolitis [34]. Moreover, hypomethylation of NF-κB-mediated pathway genes has also been conformed to contribute to COPD exacerbation [35].

The mitogen-activated protein kinase (MAPK) pathway participates in stress adaptation and inflammatory responses and its activation can stimulate cytokines, neurotransmitters, serine proteases, and oxidative stress [36]. Haemophilus influenzae is a common pathogen of COPD, and it was found to upregulate MUC gene transcription through the activation of MAPK signaling pathway [37]. In addition, IL-8 and TNF-α are key factors in COPD development and are are also regulated by p38MAPK [38].

Many other pathways including EGFR signaling pathway [39], MARCKS protein signaling pathway [40], SNARE protein signaling pathway [41], and Ciliophagy signaling pathway [42] are associated with airway mucus hypersecretion, which is recognized as one of the main pathophysiological changes in COPD patients. The cGAS-STING signaling pathway is also highly involved, and we elaborate on this pathway in this review.

## Overview of the cGAS-STING pathway

Sun et al. identified cGAS through isolation and purification in 2013 [43], and revealed a novel immune signaling pathway, namely, the cGAS-STING signaling pathway. This pathway occurs within cells and is highly important for immune systems that can sense double-stranded DNA (dsDNA) to defend against extracellular or intracellular pathogens [4]. The cGAS-STING pathway has emerged as a critical mechanism for the induction of powerful innate immune defense programs [44].

**Fig. 1:**
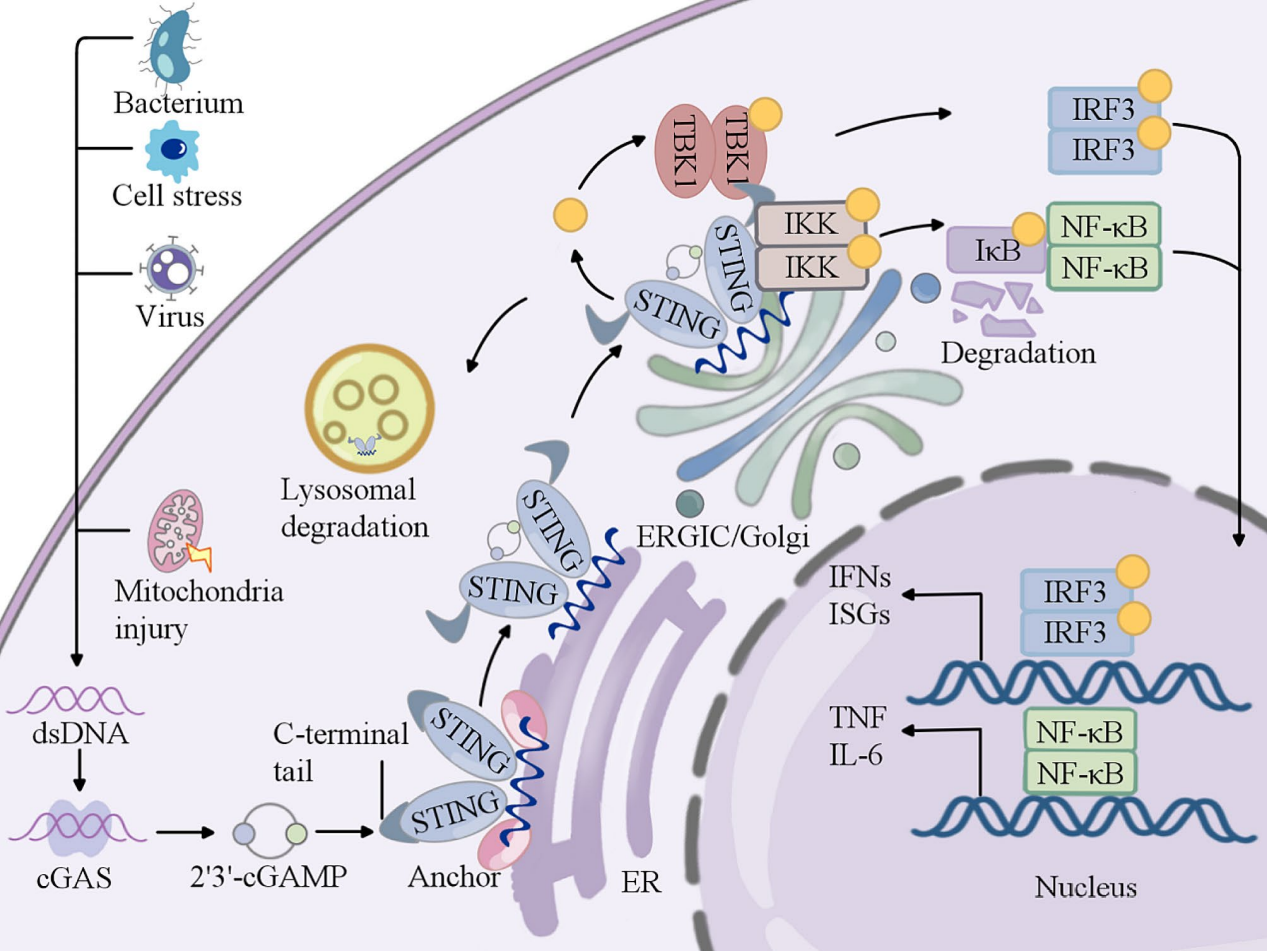
Overview of the cGAS–STING pathway

In many organisms, the detection of foreign DNA is a key factor in immunity. In mammalian cells, this process is largely facilitated by the cGAS, which has become an important mechanism for combining DNA perception with the induction of powerful innate immune defense strategies [45, 46]. PRRs are essential components of the innate immune system that can recognize biomolecules such as pathogen-associated molecular patterns (PAMPs) and DAMPs. PAMPs contain double- or single-stranded DNA and RNA generated by viral or bacterial infection or in the cytoplasm [47]. The strongest response after DNA stimulation is initiated by cGAS, a member of the PRR family, which is activated after binding to dsDNA [43] in a minimal 2:2 complex to induce conformational changes that allow cGAS to catalyze ATP and GTP into 2’,3’-cGAMP [48–50]. The sugar phosphate backbone of DNA-binds to a nucleotide transferase domain (catalytic part) in the C-terminus of cGAS, which includes positively charged DNA binding sites, a primary site, and two additional sites [45]. cGAMP binds to STING, inducing its phosphorylation of STING and causing its conformational changes, activating downstream signal transduction. During this process, STING undergoes high-order oligomerization to form tetramers [51, 52] and is transferred from the endoplasmic reticulum to the intermediate compartment of the endoplasmic reticulum Golgi apparatus. For the past several years, structural studies have shown that the tetramerization of STING in the Golgi complex is a signaling platform for recruiting and activating dimeric TANK-binding kinase 1 (TBK1) dimers through phosphorylation [53]. Conversely, TBK1 transphosphorylates the C-terminal domain of STING to recruit interferon regulatory factor 3 (IRF3) for activation [54], where IRF3 translocates to the nucleus. This gene plays a transcriptional role in the expression of immune stimulating genes (ISGs) and type 1 interferons (IFNs) [43, 50]. Moreover, STING also activated IκB kinase (IKK)-mediated induction of NF-κB-driven inflammatory genes. After activation, STING is transported to the inner lysosome for degradation, while NF-κB translocates to the nucleus, where it triggers the the expression of proinflammation cytokines(e.g., TNF and IL-6) [55, 56]. Activated STING passes through signal transduction pathways, ultimately leading to the production of a large amount of interferon and other immune related cytokines, thereby triggering an immune response. In addition, the binding of cGAS to DNA is independent of the DNA sequence [57]. Therefore, theoretically, self DNA from mitochondria or nuclei can also act as a cGAS ligand, activating the cGAS-STING pathway and triggering inflammatory responses [58]. Recent studies have also shown that endogenous cGAS is tightly bound to the nucleus and prevents its self response to its own DNA [59, 60]. Moreover, other studies have shown that cGAS inhibits homologous recombination mediated DNA repair and promotes genomic instability, micronucleus generation, and cell death under genomic stress conditions in a manner independent of STING by other studies [61]. With increasing researches on cGAS and STING, the cGAS-STING pathway has been revealed to be involved in autoimmunity, cellular senescence and inflammation inhibition, indicating that it plays an important role in the occurrence of inflammation and many diseases [6, 62, 63].(Fig. 1).

A schematic detailing the cGAS-STING signaling pathway. Upon binding dsDNA, cGAS dimers assemble on dsDNA to generate 2’-3’cGAMP. cGAMP binds to STING, leading to the translocation of STING from the ER to the Golgi and ER-Golgi intermediate compartment (ERGIC). This activation of STING recruits TBK1 and IKK, promoting their autophosphorylation and triggering the phosphorylation of IRF3 and IκB. Phosphorylated IRF3 translocates to the nucleus, where it results in the gene expression of type I interferons. Phosphorylated IκB recruits NF-κB, induces expression of genes encoding proinflammatory cytokines, and is subsequently degraded.

## The cGAS-STING pathway in COPD

### The cGAS-STING pathway in cigarette smoke-induced COPD

The significance of the cGAS-STING pathway in the inflammatory response is well known. As mentioned earlier, smoking is a key factor that induces COPD. In 2016, Pouwels revealed that exposure to a smoke environment can induce the release of dsDNA and mtDNA in mouse bronchoalveolar tissues in vivo, leading to cell death in human bronchial epithelial cells, and increased release of dsDNA and mtDNA was detected in the extracellular environment [64]. Moreover, sensing of these two types of DNA opens up the cGAS-STING immune pathway. In a 2019 study, Sears reported that exposure to CS triggers cGAS and STING expression at the mRNA and protein levels. Importantly, DNA damage repair defects are related to the pathogenesis of COPD, indicating that DNA release and sensing play a crucial role [65]. In mice and COPD patients, macrophage uptake of nanoparticulate carbon black induces DNA repair enzymes, leading to dsDNA breakage and an inflammatory response through activation of the cGAS-STING signaling pathway [66]. Moreover, in 2019, Nascimento, using gene-deficient mouse strains, reported that the absence of cGAS or STING can lead to reduced lung inflammation. In this study, the BALF of COPD model mice showed overexpression of cGAS and STING, while the DNA content increased, and neutrophil recruitment increased. Compared with wild-type mice, mice with STING and cGAS gene knockout showed a significant decrease in dsDNA content in the BALF and in the production of the downstream chemokine CXCL10 and relatively mild lung inflammation, while TLR-9 gene knockout mice showed no significant changes. This finding suggested that the self-DNA released after exposure to cigarette smoke is recognized by cGAS rather than by TLR-9, which activates the STING pathway and leads to increased secretion of type I IFN, promoting pulmonary inflammation [7]. In addition, the release of some self-dsDNA is dependent on STING, indicating that lung injury induces de novo cell death and self-dsDNA-dependent lung inflammation through amplification loops [67].

### The cGAS-STING pathway in particulate matter-induced COPD

The long-term inhalation of particulate matter, such as silica and PM 2.5, is another major cause of COPD and can also lead to chronic lung inflammation. Exposure to silica particles can induce the release of proinflammatory and profibrotic factors (e.g., IL-6, TNF-α, and TGF-β), which contribute to the acceleration of lung inflammation, and the activation of the cGAS-STING signaling pathway is involved in this process. Benmerzoug reported that mitochondria can be a source of self-dsDNA triggering DNA sensor activation after exposure to silica particles, triggering the type I IFN pathway and inducing cell death in the lungs [8]. After silica exposure, both the STING and NLRP3 pathways were activated, leading to cell death and the release of proinflammatory cytokines. This process leads to necrosis and apoptosis in a STING-dependent manner. Another study conducted by Wang in 2022 [9] revealed that PM2.5-induced aging is regulated by an inflammatory response that is activated by the cGAS/STING/NF-κB pathway, which is closely related to DNA damage. Their study also showed that pretreatment with selenomethionine (Se Met) can inhibit the inflammatory response and prevent cell aging by blocking the cGAS/STING pathway in A549 cells exposed to PM2.5. In addition, the in vivo C57BL/6J mouse model showed a decrease in cGAS expression after Se Met treatment, which can alleviate PM2.5-induced lung tissue aging in mice.

Collectively, the studies discussed here have established the implications and characteristics of STING signaling activation in COPD development. These findings suggest that known COPD-causative factors (e.g., CS, silica, and PM 2.5) can trigger the activation of the cGAS-STING signaling pathway and that targeting this pathway could help alleviate inflammation in COPD patients. Nonetheless, most of the studies discussed here were mouse model-based; therefore, there is an urgent need for human-based research to elucidate the involvement of STING in COPD.

## Antagonists of the cGAS-STING pathway

There is no cure for COPD; however, the emergence of the first drug-like compounds selectively targeting cGAS or STING has opened the door for the development of clinical candidates. Several small-molecule agonists have been developed and are being tested in tumor immunotherapy [68]. Although the cGAS-STING pathway antagonists have the optimal beneficial effects on tumor diseases, prominent efforts are underway to develop novel compounds to control the severe inflammation and acute tissue damage observed from chronic stimulation by antagonizing cGAS and STING. (Table 1)

**Table 1:** cGAS and STING antagonists

cGAS and STING Antagonists	Name	Molecular mechanism	References
cGAS inhibitors	Hydroxychloroquine	Disrupting DNA binding	[69]
	Quinacrine		[69]
	X6		[70]
	A151		[71]
	Suramin		[72]
	RU.521	Occupying cGAS Catalytic site	[73]
	PF-06928125		[74]
STING inhibitors	Tetrahydroisoquinolinone acetate (Compound 18)	Targeting the CDN-binding site	[75]
	Astin C		[76]
	Nitrofuran derivatives(C-170, C-171, etc.)	Targeting STING palmitoylation sites	[77]
Unknown inhibitors	CU-32 and CU-76	unknown mechanisms	[78]
	VS-X4		[79]

### Inhibitors of cGAS

Numerous cGAS antagonists have been identified as favorable targets for ameliorating cGAS- and STING-dependent inflammatory diseases. One example is antimalarial drugs (e.g., hydroxychloroquine, quinacrine, suramin, and oligodeoxynucleotides A151 and X6), which specifically bind to two drug sites on the 2:2 cGAS/dsDNA dimer and have the potential to suppress cGAS activity [69, 71, 72]. At each site, the antimalarial drug acts in the dsDNA minor groove between the cGAS/DNA interface (the interface connecting the dsDNA-binding site A/B on two neighboring monomers). This results in the interaction of the antimalarial drug with the DNA-binding sites (A and B) on the two neighboring cGAS monomers, which alters the stability of the cGAS/dsDNA complex and thus inhibits the activation of cGAS by dsDNA and its enzymatic activities. Follow-up studies have indicated that in the presence of the antimalarial drugs studied, IFN-β expression, cGAMP production, and the levels of a number of dsDNA-stimulated cytokines (IL-6 and TNF-α) are inhibited.

Furthermore, RU.521 affected cGAS activity by occupying the catalytic site of cGAS and decreasing its binding affinity for ATP and GTP without directly interfering with dsDNA binding, as identified in mouse studies [73]. It was demonstrated to reduce Ifnb1 mRNA expression in bone marrow-derived macrophages from Trex1-/- mice. PF-06928125 has also been shown to act as a cGAS inhibitor [74]. High-throughput biochemical screening of cGAS inhibitors identified PF-06928215. Although PF-06928215 was also able to bind to the cGAS active site with a high affinity value of 0.2 µmol/L and inhibit cGAS activity with an IC50 value of 4.9 µmol/L, PF-06928215 showed no activity in the cellular cGAS assay [80].

### Inhibitors of STING

STING is the critical signaling molecule for the cGAS–STING pathway; therefore, developing antagonists of STING may exploit cGAS–STING inhibitors. Haag et al. reported that nitrofuran derivatives, including C-170, C-171, C-176, C-178, and H-151, can block the STING-mediated signaling pathway by covalently modifying the Cys91 residue of STING [77]. Cys91 in STING has been shown to be targeted by the covalent ligand BPK-25, which inhibits STING activation by disrupting the binding of the cyclic dinucleotide ligand cGAMP [81]. Moreover, Tetrahydroisoquinolinone acetate (Compound 18) stabilizes the open, inactive conformation of STING and binds to the cGAMP binding site in a 2:1 ratio, displacing cGAMP from its binding site on STING. Compound 18 potently inhibited in vitro cGAMP-dependent signaling and displayed slow dissociation kinetics and good oral bioavailability [75]. Astin C is a natural cyclopeptide derived from the traditional Chinese medicinal plant Aster tataricus and was identified by Li et al. as a potent bioactive compound that restricts the cGAS-STING signaling to alleviate autoinflammatory response in a Trex1−/− mouse model and in macrophages [76]. It was demonstrated that astin C binds competitively to the CDN site via pull-down experiments using biotinylated astin C and human STING. Astin C blocks the recruitment of IRF3 to the STING signalosome, thus preventing downstream signaling through this pathway.

### Inhibitors of unknown mechanisms

Aside from the findings discussed above, there are also several inhibitors whose mechanisms are still unknown. For example, the small molecules CU-32 and CU-76 bind to cGAS without disrupting the binding between cGAS and dsDNA; these small molecules can inhibit the protein − protein interactions (PPIs), interfaces required for IRF3 activation and downstream IFN-I induction, in human monocyte THP-1 cells, but the exact mechanism is still unclear [78]. Additionally, the small heterocyclic compound VS-X4 has been shown to inhibit STING with no elucidated mechanism of action [79]. Further research is needed to reveal the specific mechanisms of action of these inhibitors.

### cGAS-STING inhibitors in clinical trials

Currently, some of these compounds are being phased into clinical studies. María Gómez Antúnez reported a higher survival rate in COPD patients hospitalized with SARS-CoV-2 treated with hydroxychloroquine, but they only recommend its use in clinical trials [82]. Moreover, studies has shown that Quinacrine is a potential treatment for COVID-19 virus infection [83] and that Nitrofuran is a therapy for uncomplicated lower urinary tract infection in women [84], indicating their anti-inflammatory roles. However, many of these studied inhibitors primarily treat tumors rather than COPD or other inflammatory diseases. More researches should be devoted to controlling inflammation through antagonizing the cGAS-STING pathway, and we expect that more inhibitors of cGAS-STING will enter clinical trials for COPD treatment in the future.

## Targeting the cGAS-STING signaling pathway alleviates COPD

A better understanding of the cGAS-STING signaling pathway has led to the identification of several potential therapies for inhibiting inflammation; these therapies have been termed “protectors of COPD patients”. Recent studies focused on cGAS and STING have provided new directions for treating COPD.

### Circumventing cellular senescence attenuates COPD by targeting the cGAS-STING signaling pathway

Cellular senescence is a state of cell cycle arrest and is among the 9 hallmarks of aging proposed by López-Otín in a landmark paper [85]. Senescent cells including alveolar epithelial and endothelial cells accumulate in the lungs COPD patients [86, 87], and CS-induced oxidative stress is likely to play an important role in the induction of senescence in COPD [88]. In a mouse model-based study, Takao demonstrated the induction of senescence in lung parenchymal cells during the progression of COPD [89].And evidence from clinical samples of primary human bronchial epithelial cells and lung homogenates from COPD patients indicates the same conclusion [90].

Studies have shown that interfering with DNA binding to the DNA sensor cGAS can induce cellular senescence [10, 11]. Aging-accelerated factors induce the proinflammatory Senescence Associated Secretory Phenotype (SASP), leading to leakage of DNA into the cytoplasm and triggering of the cGAS-STING pathway of the innate immune response [12]. The cGAS-STING also induces the SASP phenotype by accumulating cytoplasmic DNA during senescence [10, 91], thus aggravating the aging response. Wang et al. reported that Se-Met treatment prevents PM2.5-induced senescence via attenuating inflammatory response regulated by cGAS/STING/NF-κB pathway, and further causes a reduction in COPD [9]. Since cGAS-STING pathway plays important roles in COPD, these studies further indicate that targeting this pathway may circumvent cellular senescence and thus has therapeutic potential for mitigating COPD.

### Natural products relieve COPD by targeting the cGAS-STING signaling pathway

The Tanreqing (TRQ) injection is a Chinese patent medicine. It can significantly improve the partial pressure of oxygen (PaO2), partial pressure of carbon dioxide (PaCO2) and lung function in patients with COPD combined with respiratory failure, and is commonly used to treat AECOPD. Several in vivo experimental studies have also revealed that TRQI can reduce the expression of IL-8, TNF-α and mucin 5AC (MUC5A) in alveolar lavage fluid in CS-and LPS-induced rats with COPD, thereby improving the inflammatory response of airway mucosa and inhibiting airway mucus hypersecretion in rats [92]. TRQ injection inhibited STING levels, suggesting that TRQ has therapeutic efficacy by blocking the increase in the cGAS-STING pathway in COPD patients [93]. However, the specific mechanism of action of TRQI for the treatment of COPD is still unclear. With respect to the five traditional Chinese medicines, further experiments are needed to identify the specific components of TRQ that regulate the cGAS-STING pathway and alleviate COPD.

Panax ginseng C.A Meyer (ginseng) root is another important traditional Chinese medicinal herb. Its pharmacologically active constituents have been identified, most notably ginsenosides, which are triterpenoid saponins that include protopanaxadiol (PPD) and protopanaxatriol (PPT) [94]. The PPD group contains ginsenosides Rb1, Rb2, Rb3, Rc, Rd, Rg3 and Rh2 and compound K, while the PPT group comprises ginsenosides Re, Rf, Rg1, Rg2 and Rh1. These compounds possess pharmacological effects, such as antiviral, antioxidant, and immunomodulatory activities, and have potentially relevant effects on COPD, including the inhibition of proinflammatory mediators and cytokines [95]. Recently, studies have shown that ginsenosides can alleviate COPD and reduce lung injury [96, 97]. In a CS-induced BALV/c mouse model, X. Guan et al. reported that ginsenosides Rg3 and Rb3 could negatively regulate PI3K activation, NF-κB activity, and proinflammatory cytokines in a CS-induced BALV/c rat model, basal cells, and a coculture model of bronchial epithelial cells and neutrophils, thus reducing neutrophil migration. Moreover, ginsenosides basically inhibit various COPD-related pathogenesis processes, such as inflammatory responses (TNF-α, IL-6, IL-1β, NF-κB induction and translocation), kinase phosphorylation (MAPK and ERK1/2), and oxidative stress (ROS) [98]. Mechanistically, PPD suppressed the cGAS-SING pathway through the activation of AMPK and the inhibition of TNF, IL and NF-κB. The therapeutic effect of PPD in COPD patients awaits further clinical investigation.

Both Radix Pseudostellariae and Juglanin, which are types of tonic Chinese medicine, have been shown to reduce lung inflammation by inhibiting the cGAS-STING pathway and therefore alleviating COPD [99, 100]. The findings above suggest that natural products, especially Chinese medical herbs, can alleviate inflammation by inhibiting the cGAS-SING pathway, thus exerting therapeutic effects on COPD. This finding provides potential avenues for future drug development and therapeutic strategies for this disease.

## Discussion: summary, outstanding questions, and future directions

Airway inflammation is a consistent characteristic of COPD and is related to its pathogenesis and progression. As an innate immune pathway, cGAS-STING plays an undeniable role in immune-related diseases. The cGAS-STING pathway has been shown to alleviate inflammatory responses and lung function damage in COPD patients, indicating its potential as a therapeutic target [7]. This pathway offers a specific and selective means to modulate immune responses, particularly in DNA-induced inflammation associated with COPD. Current preclinical development efforts are focused on several cGAS and STING inhibitors, including antimalarial drugs [77], RU.521 [73], PF-06928215 [74], nitrofuran [77], compound 18 [75] and astin C [76], ect., which are expected to open up new avenues for treating COPD. Furthermore, targeting the cGAS-STING signaling pathway circumvents cellular senescence to attenuate COPD [10, 11] and an increasing number of natural products, mainly Chinese herbs, have been discovered to alleviate COPD via a pharmacological mechanism involving cGAS-STING [93, 98–100].

However, the pathogenesis of COPD is not fully understood. Most relevant studies are animal-based experiments rather than human-based ones, which makes their applicability in humans a question. Although the cGAS-STING pathway is highly involved in the inflammatory mechanism of COPD, it is merely a fraction of the overall picture, necessitating further exploration of additional mechanisms. Moreover, inhibitors of the cGAS-STING signaling pathway have not been extensively employed as COPD therapeutic drugs, and the mechanisms by which certain drugs function as inhibitors remain unclear.

In this review, we discuss the inflammatory pathogenesis of COPD and provide an overview of the current understanding of the cGAS-STING signaling pathway as well as its potential as a COPD therapeutic target. The cGAS-STING pathway is activated in COPD, and its activation further exacerbates the development of COPD. Targeting the cGAS-STING signaling pathway is highly important for curing COPD, and many studies have suggested that small molecule inhibitors are effective controlling the development of COPD. However, whether COPD is related to cGAS and STING remains an area that requires further research. In conclusion, targeting the cGAS-STING signaling pathway provides a promising direction for COPD therapy and intervention.

## Data Availability

No datasets were generated or analysed during the current study.

## Abbreviations

COPD: Chronic Obstructive Pulmonary Disease
cGAS: cyclic GMP-AMP Synthase
STING: Stimulator of Interferon Genes
CS: Cigarette Smoking
PRRs: Pattern Recognition Receptors
cGAMP: cyclic GMP-AMP
ER: Endoplasmic Reticulum
GOLD: The Global Initiative of Obstructive Lung Disease
DAMPs: Damage-Associated Molecular Patterns
IL: Interleukin
TSLP: Thymic Stromal Lymphopoietin
ILC: Innate Lymphoid Cells
MCP: Monocyte Chemotactic Proteins
CCL: C-C motif chemokine ligand
dsDNA: double-stranded DNA
PAMPs: Pathogen Associated Molecular Patterns
DAMP: Damage Associated Molecular Pattern
TBK1: TANK-binding Kinase 1
IRF3: Interferon Regulatory Factor 3
ISGs: Immune Stimulating Genes
IFNs: Interferons
IKK: IκB Kinase
ERGIC: ER-Golgi Intermediate Compartment
Se Met: Selenomethionine
PPI: Protein − protein Interface
SASP: Senescence Associated Secretory Phenotype
TRQ: Tanreqing
PaO2: Partial Pressure of Oxygen
PaCO2: Pressure of Carbon Dioxide
MUC5A: Mucin 5AC
Ginseng: Panax Ginseng C.A Meyer
PPD: Protopanaxadiol
PPT: Protopanaxatriol
ROS: Reactive Oxygen Species
